# Histoplasmosis in Children; HIV/AIDS Not a Major Driver

**DOI:** 10.3390/jof7070530

**Published:** 2021-06-30

**Authors:** Bassey E. Ekeng, Kevin Edem, Ikechukwu Amamilo, Zachary Panos, David W. Denning, Rita O. Oladele

**Affiliations:** 1 Department of Medical Microbiology and Parasitology, University of Calabar Teaching Hospital, Calabar 540271, Nigeria; 2 Department of Paediatrics, University of Uyo Teaching Hospital, Uyo 52021, Nigeria; edemkevin@gmail.com; 3 Clinton Health Access Initiative, Department of Infectious Diseases Global HIV Access Program, Abuja 900287, Nigeria; iamamilo@clintonhealthaccess.org; 4 Clinton Health Access Initiative, Department of Infectious Diseases Global HIV Access Program, Washington, DC 20560, USA; zpanos@clintonhealthaccess.org; 5 Global Action Fund for Fungal Infections, 1211 Geneva, Switzerland; ddenning@gaffi.org; 6 Department of Medical Microbiology and Parasitology, Faculty of Basic Medical Sciences, College of Medicine, University of Lagos, Lagos 100254, Nigeria

**Keywords:** histoplasmosis, children, low medium countries, HIV, childhood malignancies, immunosuppressants

## Abstract

The classification of histoplasmosis as an AIDS-defining illness has largely attributed its occurrence in people to the presence of HIV/AIDS especially in Africa. Prior to the advent of the HIV/AIDS epidemic, many cases of histoplasmosis were documented both in the pediatric and adult population. Our review revealed 1461 reported cases of pediatric histoplasmosis globally in the last eight decades (1939–2021). North America (*n* = 1231) had the highest number of cases, followed by South America (*n* = 135), Africa (n = 65), Asia (*n* = 26) and Europe (*n* = 4). Histoplasmosis was much more common in the non-HIV pediatric population (*n* = 1418, 97.1%) compared to the HIV population. The non-HIV factors implicated were, childhood malignancies (*n* = 207), such as leukemias and lymphomas as well as their treatment, lung diseases (*n* = 7), environmental exposures and toxins (*n* = 224), autoimmune diseases (*n* = 12), organ transplants (*n* = 12), long-term steroid therapy (*n* = 3), the use of immunosuppressive drugs such as TNF-alpha inhibitors (*n* = 7) malnutrition (*n* = 12), histiocytosis (*n* = 3), hyperimmunoglobulin M and E syndromes (*n* = 15, 1.2%), pancytopaenias (*n* = 26), diabetes mellitus (*n* = 1) and T-cell deficiency (*n* = 21). Paediatricians should always consider or rule out a diagnosis of histoplasmosis in children presenting with symptoms suggestive of the above clinical conditions.

## 1. Introduction

Histoplasmosis is a systemic fungal infection occurring globally. It is endemic in the Ohio and Mississippi river valleys of the United States of America, India and Southeast Asia [1,2]. The clinical presentation of histoplasmosis mimics features seen in tuberculosis (TB) and may be misdiagnosed as such [3]. Amongst risk factors associated with disseminated histoplasmosis (DH), HIV infection has been attributed as the greatest factor predisposing patients to DH in the adult population [4]. However, before the advent of HIV/AIDS, several cases of DH had been reported [5]. Additionally, a review on the documented cases of histoplasmosis in Africa, from 1952–2017 by Oladele et al. revealed 37 cases of histoplasmosis in the pediatric age group, of which 33 (89.2%) were non-HIV patients [4]. In another report by Arango et al. of the Colombian National Survey, 1992–2008, 17 cases of histoplasmosis were reported within the pediatric age group, two were patients with AIDS, four had hematological cancers, three had environmental exposures, while eight had unknown risk factors [6]. In yet another study on childhood histoplasmosis by Lopez et al. from 1984 to 2010, of the total 45 cases found, only four cases were HIV positive patients [7]. The other cases were attributed to malnutrition, tumors and environmental exposure [7]. Pakasa et al., in a case series study also revealed a high incidence (41.744.4%, 15/36) of histoplasmosis in non-HIV children within the age range of 3–16 years [8]. A large percentage of histoplasmosis in the pediatric age group most likely occurs in the non-HIV population, despite being described as an AIDS-defining illness. The purpose of this review is to identify factors other than HIV infection predisposing children to histoplasmosis and to raise awareness on pediatric histoplasmosis in the non-HIV population.

## 2. Mycology Summary

Histoplasmosis is caused by the dimorphic fungus, *Histoplasma capsulatum.* The two varieties pathogenic to humans are *H. capsulatum* var. *capsulatum* and *H. capsulatum* var. *duboisii*. The third variety, *H. capsulatum* var. *farciminosum,* is an equine pathogen [2]. However, a new taxonomic rearrangement of *H. capsulatum* proposed by Sepúlveda VE et al. showed that it is composed of at least four different cryptic species that differ genetically and also in virulence: *Histoplasma capsulatum* (Panama or H81 lineage), *Histoplasma mississippiense* (NAm 1), *Histoplasma ohiense* (NAm 2) and *Histoplasma suramericanum* (LAm A) [2]. Human infection occurs by inhalation of microconidia and short hyphal fragments which are converted into yeast forms in the alveoli. Transmission can also be orally, causing infection in the intestines [9]. Transplacental, congenital and perinatal transmission of infections have also been reported [10,11,12,13,14]. Histoplasmosis is primarily a pulmonary disease but can be disseminated in individuals at the extremes of ages due to their weak immune system, in people with advanced HIV/AIDS disease and secondary to persistent exposures to *Histoplasma* conidia [2,15]. Extrapulmonary presentation involving the skin may also occur and may progress to cause bone damage [3].

## 3. Materials and Methods

Literature searches for publications on histoplasmosis in children preceding February 2021 were performed using PubMed, Google Scholar, AJOL, Cochrane Library, Africa-Wide: NiPAD, CINAHL (accessed via EBSCO Host) databases and grey literature to identify all published papers regarding the topic. The main search comprised individual searches using detailed medical subject heading terms for histoplasmosis in children, including broad terms such as “case reports of histoplasmosis in children” and/or ‘diagnosis’ and ‘management’ of histoplasmosis in children. References in all relevant papers were also reviewed for additional publications (‘snow balling’) on histoplasmosis in children that may not have been published in the searched databases. No language restriction was applied. Only reports with patients’ country of origin identified were included. Publications reporting cases of histoplasmosis in individuals greater than 18 years of age were excluded from the review, as well as studies involving histoplasmosis in non-humans. Publications without abstracts were also excluded. The case definitions employed were based on an international consensus statement by the European Organization for Research and Treatment of Cancer/Invasive Fungal Infections Cooperative Group (EORTC/IFICG) and the Mycoses Study Group (MSG) [16]. Case reports with undefined HIV status were assumed to be negative while case reports with defined HIV status (that is positive or negative) were documented as such.

## 4. Results

**Figure 1:**
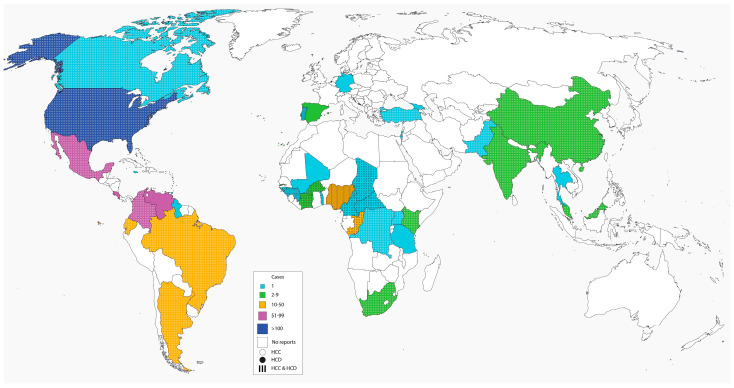
Global distribution of histoplasmosis in children.

Histoplasmosis in children has been reported globally with high endemicity in Northern and Southern America [5,17]. Our literature search revealed a total of 1461 documented cases of histoplasmosis in children reported globally (Table 1) dating from 1945 to 2021. The number of cases were highest in North America (*n* = 1231), with 76.7% (944/1231) occurring in the USA. Very few cases (*n* = 4) in the pediatric population have been reported in Europe and were found in immigrants from endemic regions [18,19,20]. The number of documented cases from other climes were, South America (*n* = 135), Africa (*n* = 65) and Asia (*n* = 26) respectively. More cases occurred in the non-HIV population compared to the HIV population, globally. The percentages of non-HIV patients with pediatric histoplasmosis across the various regions was 97.1% (1418/1461) and North America (96.8%, 1192/1231), South America (99.3%, 134/135), Africa (98.5%, 64/65), Asia (96.2%, 25/26) within each region, respectively, Table 1, Figure 1. Pediatric histoplasmosis in America and China were caused by *Histoplasma capsulatum* var *capsulatum* (Hcc), while in Africa, it was largely due to *Histoplasma capsulatum* var *duboisii* (Hcd) (88.7%, 47/53). Most cases of disseminated histoplasmosis occurred in persons with compromised immunity (Table 2) [21,22]. These infections are also common at the extremes of age, i.e., in infants and the elderly, although cases of disseminated histoplasmosis have been reported in apparently normal individuals without the usual risk factors of extremes of age or some form of immunosuppression [23,24]. Childhood cancers such as leukemias and lymphomas as well as their treatment may predispose to histoplasmosis [21]. Underlying lung diseases, environmental exposures and toxins, autoimmune disease, long-term steroid therapy, use of immunosuppressive drugs such as TNF-alpha inhibitors may also predispose to histoplasmosis in children who are HIV-negative. Other less common conditions include histiocytosis, hyperimmunoglobulin M and E syndromes and pancytopaenias (Table 1, Table 2 and Table 3) [21,22]. DH has also been documented in both premature and mature infants with variable outcomes [25,26,27,28,29].

## 5. Discussion

### 5.1. Pulmonary Histoplasmosis and TB

Histoplasmosis may be misdiagnosed as TB due to the similarities in their clinical presentation [3]. Mosam et al., 2006 reported a case of disseminated histoplasmosis in an 11-year-old female African child who initially received anti-Koch’s regimen for two weeks despite negative Mantoux test and negative AFB [3]. Additionally, though rare in the literature, cooccurrence of histoplasmosis and TB has also been documented in the pediatric age group [150]. Clinicians should investigate more with respect to histoplasmosis when seeing this cohort of patients.

### 5.2. Cancers

Pediatric cancers are probably the commonest predisposing factor to histoplasmosis infection in non-HIV patients. Conversely disseminated histoplasmosis can present with pancytopenia, and be initially mistaken for leukaemia or aplastic anaemia. The presentation may be localized i.e., pulmonary disease or may be disseminated although the latter appears to be commoner [21,61]. Most children have an underlying hematologic malignancy (leukemia or lymphoma) and the diagnosis of histoplasmosis may be challenging due to the similarity in appearance of pulmonary histoplasmosis to lung metastases of primary malignancies [21]. A lung biopsy may be required to distinguish the two [22]. The impairment of T-cell function in malignancies is the pathogenetic mechanism for the increase in susceptibility to histoplasmosis in pediatric cancer patients [35,36]. The use of chemotherapeutic agents resulting in bone marrow depression and consequently predisposing the children to histoplasmosis and other opportunistic infections is another pathogenetic explanation for the increased incidence [21]. A 14-year review of 57 pediatric cancer patients by Adderson revealed that there were 61 episodes of histoplasmosis in the group with disseminated histoplasmosis being more common [61]. Fever was the most common presenting symptom and no mortalities were attributable to histoplasmosis but cancer therapy was often delayed or modified due to the infection. Most patients initially received unnecessary antibiotic therapy and they were mostly non-neutropenic. Hess et al. found that urine antigen detection was highly sensitive for disseminated disease in oncology patients but lacked sensitivity in patients with acute pulmonary disease and if utilized alone may lead to missed diagnoses [21].

### 5.3. Lung Diseases

Pediatric histoplasmosis most frequently manifests as an acute pulmonary disease [57]. In adults, pulmonary histoplasmosis affect individuals with underlying lung disease such as emphysema and less commonly chronic bronchitis [151]. Pulmonary histoplasmosis has also been reported in individuals previously diagnosed with or treated for tuberculosis [151]. Asthma, the commonest chronic lung disorder of childhood is thought to predispose to pulmonary histoplasmosis with possible pathogenetic mechanisms including alterations in local immunity of the respiratory tract and chronic use of corticosteroid treatments [181]. The differential diagnosis should be considered in children with asthma who present with recurrent or prolonged symptoms and who have been exposed to Histoplasma, as endemic areas and bat caves [181].

### 5.4. Environmental Exposure

Most cases of histoplasmosis in healthy children result in a clinically inapparent infection. Immunocompromised hosts, infants and the elderly may present with clinical and often disseminated disease [113]. The size of the inoculum which is a function of the degree of exposure is also implicated in the tendency to develop clinical infections. Daubenton et al. reported a case of a seven-year-old immunocompetent child who lived on a poultry farm and developed disseminated histoplasmosis [23]. Although the relevant environmental exposure had been exposure to bat caves, non-cave exposures werehave also been reported [23]. For instance, Haselow et al. reported that 12 children age range 4–19 years had contracted histoplasmosis after attending a family bonfire which used bamboo retrieved from a large red blackbird roost [90]. Seven of the children were ill enough to require admission but there were no reported fatalities. The large ‘inoculating dose’ and the age of the children were probably important factors in the spread of the disease in the case.

### 5.5. Malnutrition

Malnutrition is strongly associated with pediatric morbidity and mortality. The World Health Organization reported in 2020 that malnutrition was a contributing factor in 45% of all mortalities in under-five children worldwide [184]. Malnutrition is the primary cause of immunodeficiency worldwide and young children and the elderly are most affected [185]. Malnutrition leads to defects in both innate and acquired immunity and sets off a vicious cycle with malnutrition leading to poor immunity and increased risk of infections/more severe infections which will then lead to poor intake, increased metabolic demand for nutrients leading to even more malnutrition. Defects in cell-mediated immunity caused by malnutrition also predispose to histoplasmosis. Mata-Essayag et al., in a retrospective review of 158 cases of histoplasmosis in Venezuela noted that 6 out of 42 children (14.3%) with histoplasmosis were also malnourished [130]. Of these, four (67%) died [130]. Lopez et al., in a study of 45 Colombian children with histoplasmosis found that malnutrition was the most important risk factor being found in 37% of the patients [7]. In an earlier study by Jimenez et al., malnutrition was an association in over two-thirds of children with disseminated histoplasmosis. The mortality rate in the study was 61% [41].

### 5.6. Organ Transplant

Organ transplant recipients are susceptible to infections due to the chronic use of immunosuppressive therapy [92]. While viral infections are far commoner in solid organ transplant recipients, fungal infections are usually more serious [50]. Studies have reported on occurrences of disseminated histoplasmosis in pediatric kidney transplant patients which are by far the commonest solid organ transplants done in the pediatric age group [92,94]. Hemophagocytic lymphohistiocytosis has often been reported in association with histoplasmosis infection in kidney transplant recipients and this complicates the management and often worsens the prognosis [50,92,94]. A case series of six pediatric kidney transplant patients in an endemic region of the USA put the attack rate of histoplasmosis among kidney transplant patients at 6.9/100 cases. One-third of the cases occurred in the first year following the kidney transplant and there were no fatalities [94]. Most of the patients were treated with amphotericin B and then transitioned to an azole, with 83% receiving chronic suppression therapy with itraconazole to prevent future occurrences, such therapy was usually given indefinitely. The authors suggested there may be a case for histoplasmosis prophylaxis for pediatric transplant recipients in endemic areas [94].

### 5.7. Autoimmune Diseases/Steroid Therapy

Pediatric autoimmune diseases like juvenile rheumatoid arthritis (JRA) are frequently treated with steroids and other immunosuppressive agents including methotrexate. Because of the low doses used, opportunistic infections are not very common in association with autoimmune steroid or immunosuppressive therapy [102]. Hunstad et al. reported a case of a seven-year-old female with JRA who developed histoplasmosis while on low-dose methotrexate. She had also recently been weaned off prednisolone treatment [102]. The authors also noted that histoplasmosis and other opportunistic infections (OIs) were less common in JRA patients receiving low-dose methotrexate than in adult patients receiving the same therapy for rheumatoid arthritis. The rarity of fungal opportunistic infections including histoplasmosis has also been noted among pediatric patients with systemic lupus erythematosus (SLE) with the first case report of Histoplasmosis diagnosed in a child with SLE being as recent as 2012 [121]. The patient in question presented with abdominal distension, splenomegaly and multiple mesenteric lymph nodes and unfortunately died of septic shock while undergoing treatment. While these reports are rare, the increasing number of children being diagnosed with autoimmune disorders and on treatment with steroids and immunosuppressive agents makes a case for pediatricians and rheumatologists to be vigilant in identifying such cases so outcomes can be improved.

### 5.8. Pancytopaenia/Defective Phagocytic Function

Pancytopaenia and defective phagocytic function usually in association with haemophagocytic lymphohistiocytosis (HLH) has been noted as a predisposing factor to pediatric histoplasmosis with Russ et al. reporting a case of histoplasmosis and HLH in a seven-month-old infant with vomiting and failure to thrive [186]. The report noted that the child lacked homozygosity for HLH or had an underlying immunodeficiency, and suggested that alterations in T-cell function increased her risk for HLH following the infection with histoplasmosis, although it was unclear which condition occurred first [186]. Children with defective T- and B-cell function as well as a defective phagocytic function also present with fungal opportunistic infections such as disseminated histoplasmosis. Such reports are not common in literature and are mostly from the developed world, possibly due to better diagnostic facilities. Hasliza et al. reported a case of disseminated histoplasmosis in a two-year-old previously healthy child. The workup during the illness identified gross leucopenia with defective T-cell and phagocyte function [179]. The T-cell and phagocyte function were all found to have normalized three months after the illness.

### 5.9. Tumor Necrosis Factors Inhibitors

Tumor necrosis factor-alpha (TNF-alpha) inhibitors such as adalimumab, infliximab and etanercept are commonly used in the treatment of pediatric inflammatory bowel disease [49,78]. They may also be used as second-line agents for auto-immune disorders of childhood such as JRA and psoriasis. However, inhibition of the TNF-alpha pathway results in a predisposition to severe infections including histoplasmosis, which is the commonest invasive fungal infection in patients on TNF-alpha therapy [78]. Dotson et al. reviewed five cases of histoplasmosis in children receiving either infliximab or adalimumab for Crohn’s disease and noted that early diagnosis with discontinuation of anti-TNF medication as well as close follow-up were key to achieving good outcomes [49]. The study did not however recommend screening for latent histoplasmosis before commencing anti-TNF alpha drugs. Another report in an eight-year-old male with Crohn’s disease on Infliximab who presented with histoplasmosis and concurrent Pneumocystis pneumonia further raised the issue of pre-commencement screening for opportunistic infections in children receiving TNF-alpha inhibitors [49].

### 5.10. Syndromes (Hyperimmunoglobulin E Syndrome, Hyperimmunoglobulin M Syndrome)

Hyperimmunoglobulin E syndrome also known as Job’s syndrome and hyperimmunoglobulin M syndrome are associated with defects in immune function specifically with defects in immunoglobulin levels, neutrophil chemotactic defects, defects in T-cell signaling and cytokine abnormalities [74,83]. Impaired Th 17 cell differentiation is an immune defect consistently found in the STAT3 Job’s syndrome mutation and this mutation impairs protection against invasive fungal infections including histoplasmosis [74,83]. Case reports from pediatric patients have shown that both syndromes are associated with pulmonary, disseminated [74,83], and even cutaneous histoplasmosis infection [184].

## 6. Conclusions

This study has revealed the high occurrence rate of histoplasmosis in the non-HIV pediatric population, a disease commonly described as an AIDS-defining infection. We have also documented the underlying condition more commonly associated with this disease in children. Misdiagnosis of histoplasmosis as tuberculosis due to their similar pattern of presentation has also been emphasized. Physicians managing children with risk factors highlighted in this study and presenting symptoms suggestive of TB should be screened to exclude or include histoplasmosis.

## Figures and Tables

**Table 1:** Global distribution of Pediatric histoplasmosis.

Country	n	−HIV	+HIV	Hcc	Hcd	Factors Other than HIV (Number)	None	References
**NORTH AMERICA**								
Canada	1	-	1	1	-	LD (1)	-	[30]
* Colombia	73	69	4	25	-	Leu/Can (5), OT (2), TNFi (1), EE (9), Mal (10)	42	[6,7,31,32,33,34]
* Costa Rica	84	84	-	-	-	EE (44), TD (20), HIg (10)	10	[35,36]
Ecuador	38	14	24	37	-	HIg (1)	13	[37,38,39]
Jamaica	1	-	1	1	-	-	-	[40]
* Mexico	89	85	4	15	-	AIC (3)	82	[41,42,43,44]
Trinidad	1	-	1	1	-	-	-	[45]
* USA	944	940	4	296	-	LD (5), Mal (1), EE (163), OT (6), HIg (3), Leu/Can (198), Pan (22), IRD (1), TNFi (6), AIC (8), HLH (3), Steroids (2), TD (1)	521	[17,21,22,25,26,27,28,46,47,48,49,50,51,52,53,54,55,56,57,58,59,60,61,62,63,64,65,66,67,68,69,70,71,72,73,74,75,76,77,78,79,80,81,82,83,84,85,86,87,88,89,90,91,92,93,94,95,96,97,98,99,100,101,102,103,104,105,106,107,108,109,110,111,112,113,114,115,116]
**Total**	**1231**	**1192**	**39**	**376**	**-**	**525**	**668**	
**SOUTH AMERICA**								
* Argentina	50	50	-	11	-	EE (1)	49	[117,118,119]
* Brazil	27	26	1	5	-	EE (5), Leu/Can (2), OT (1), SLE/Steroids (1), DM (1)	16	[120,121,122,123,124,125,126,127,128]
Guyana	1	1	-	-	-	OT	-	[129]
* Venezuela	57	57	-	-	-	-	57	[130]
**Total**	**135**	**134**	**1**	**16**	**-**	**12**	**122**	
**# EUROPE**								
Germany	1	1	-	-	-	-	1	[18]
Portugal	1	1	-	-	1	-	1	[19]
Spain	2	2	-	-	-	Pan (2)	-	[20]
**Total**	**4**	**4**	**-**	**-**	**1**	**2**	**2**	
**AFRICA**								
Burkinafaso	3	3	-	-	3	-	3	[131,132]
Cameroon	1	1	-	-	1	-	1	[133]
CAR	1	1	-	-	1	-	1	[134]
Chad	1	1	-	-	1	-	1	[135]
Congo	26	26	-	-	26	-	26	[136,137,138,139,140,141,142,143,144,145]
Cote d′voire	4	3	1	-	4	-	3	[146,147,148]
Gambia	3	3	-	-	3	-	3	[149]
Guinea-Bissau	1	1	-	-	1	-	1	[150]
Kenya	2	2	-	2	-	-	2	[151]
Mali	1	1	-	-	-	EE	-	[152]
Nigeria	13	13	-	2	7	Leu/Can (2), Mal (1), OT (1)	9	[153,154,155,156,157,158,159,160,161]
South Africa	5	5	-	2	-	EE (1)	4	[23,162,163,164,165]
Tanzania	1	1	-	-	-	-	1	[166]
Togo	1	1	-	-	1	-	1	[167]
Uganda	1	1	-	1	-	MC	-	[168]
Zaire	1	1	-	1	-	-	1	[169]
**Total**	**65**	**64**	**1**	**8**	**48**	**7**	**57**	
**ASIA**								
China	9	8	1	1	-	-	8	[170,171,172]
Georgia	1	1	-	-	-	-	1	[173]
India	5	5	-	4	-	Pan (1)	4	[24,174,175,176,177]
Israel	1	1	-	1	-	LD	-	[178]
Malaysia	7	7	-	1	-	Pan (1)	6	[179,180]
Pakistan	1	1	-	1	-	-	1	[181]
Thailand	1	1	-	-	-	OT	-	[182]
Turkey	1	1	-	1	-	HIg	-	[183]
**Total**	**26**	**25**	**1**	**9**	**-**	**5**	**20**	
**Grand total**	**1461**	**1419**	**42**	**409**	**49**	**551**	**869**	

n, number of cases; HIV, Human immunodeficiency virus; Hcc, *Histoplalsma capsulatum* var *capsulatum*; Hcd, *Histoplasma capsulatum* var *duboisii;* LD, Lung diseases; Leu/Can, Leukemia and Cancers; DM, Diabetes mellitus; OT, Organ transplant; TNFi, Tumor necrosis factor inhibitors; EE, Environmental exposures; Mal, Malnutrition; TD, T cell deficiency, HIg, Hyperimmunoglobulinemia; IRD, Interferon-gamma Receptor-1 Deficiency; hemophagocytic lymphohistiocytosis (HLH); Pan, Pancytopenia; MC, mesenteric cyst: CAR, Central African Republic; # all documented case reports were in patients that immigrated from endemic regions * Majority of diagnoses were based on serological data.

**Table 2:** Percentages of non-HIV factors predisposing to pediatric histoplasmosis.

Factors	Number of Cases	%
Environmental exposure	224	15.8
Leukaemia/Cancers	207	14.6
Pancytopaenia	26	1.8
T cell deficiency	21	1.5
Hyperimmunoglobulinemia	15	1.1
Organ transplant	12	0.8
Malnutrition	12	0.8
Tumour necrosing factor inhibitors	7	0.5
Lung diseases	7	0.5
Autoimmune conditions (SLE, Rheumatoid arthritis)	11	0.8
hemophagocytic lymphohistiocytosis	3	0.2
Steroids	3	0.14
Interferon-gamma Receptor 1 Deficiency	1	0.07
Mesenteric cyst	1	0.07
Diabetes mellitus	1	0.07
**Total**	**551**	**38.8%**

% = number of cases/total number of non-HIV cases (1419).

**Table 3:** Some case reports on pediatric histoplasmosis associated with non-HIV factors.

Authors	Case Patients (n)	Age/Sex/Country	Non-HIV Factors	Clinical Findings	Diagnostic Method	Treatment and Clinical Course	OC
Hess et al., 2017	1	11-year-old/Male/USA	T-cell ALL	fever, malaise, and weight loss	CT scan: mediastinal adenopathy. Bone marrow: pancytopenia, noncaseating granulomas. Urine and serum histoplasma antigen was positive. Histology of bone marrow aspirate was in keeping with DH	AmB therapy. Histoplasma antigen in urine and serum was undetected after 3 months of therapy.	R
Tu et al., 1991	2	3-year-old/Male/USA	hyper-lgM syndrome	fever, nausea, vomiting, stomatitis of the gingiva, abdominal pain, fatigue, and irritability.	Disseminated histoplasmosis was diagnosed by bone marrow biopsy and culture of esophageal biopsy	Amphotericin B therapy	NR
Yilmaz et al., 1995	3	3-year-old/Male/Turkey	Hyper-IgM syndrome	Recurrent pulmonary infections, ulcerated lesion of about 10 cm on the left side of the face	IgM, 1380 mg/dl. Chest radiograph showed bilateral infiltration and bronchiectasis. Biopsy of skin lesion revealed intracellular yeast cells	ketoconazole 10 mg/kg/day, with immunoglobulin therapy	R
Hess et al., 2017	4	14-Year-old/Male/USA	Desmoplastic small round cell tumor of the abdomen	Persistent fever and neutropenia	Chest CT demonstrated a solitary calcified lung nodule. Histoplasma serology became positive with increasing titers of histoplasmosis yeast by complement fixation from 1:16 to 1:32. Diagnosis was pulmonary histoplasmosis	Itraconazole, Posaconazole and AmB were used for therapy	NR
Tschudy et al., 2010	5	8-year-old/Male/USA	Crohn Disease/Infliximab therapy	fevers, cough, anorexia, and night sweats.	Urine histoplasma antigen test: positive. Fungal culture grew *Histoplasma capsulatum* from lymph node biopsy. Histoplasmosis antibodies to both yeast and mycelia returned elevated (1:1024 yeast and 1:16 mycelia).	Itraconazole (6 mg/kg/day, divided twice per day)	R
Zerbe et al., 2005	6	3 years and 8 months/Male/USA	Interferon-g Receptor 1 Deficiency	Recurrent fever, hepatosplenomegaly, left cervical adenopathy	Bronchoscopic washings, a sputum specimen, and a bone marrow aspirate all grew *H. capsulatum*. Genetic studies showed 818del4 deletion in IFNGR1, which leads to autosomal dominant IFN-gamma receptor deficiency	Subcutaneous treatment with IFN-gamma (50 µg/m^2^) was given 3 times/week. Oral ketoconazole therapy	R
Hess et al., 2017	7	6-year-old/Male/USA	B-Cell ALL	nonspecific fever	Blood culture was positive for *Histoplasma capsulatum*. Chest CT: right hilar adenopathy. Antigenuria and antigenemia were confirmed. Diagnosis was DH	AmB for 14 days followed by itraconazole.	R
Hess et al., 2017	8	8-year-old/Male/USA	Stage II abdominal Burkitt lymphoma	Fever and neutropenia	Histoplasma serology was positive. Diagnosis was pulmonary histoplasmosis.	Itraconazole therapy	R
Ferguson-Pau et al., 2018	9	18-year-old/ Female/ USA	Kidney transplant	Fever, chest pain, weight loss, malaise, headache, and myalgias post-renal transplant	Urine histoplasma antigen was positive at 10.69 ng/mL	Itraconazole therapy	R
Ferguson-Pau et al., 2018	10	16-year- old/Female/USA	Kidney transplant	Fever, vomiting, diarrhea and weight loss.	Pericardial fluid and bronchoalveolar lavage were histoplasma antigen-positive. Both serum and urine histoplasma antigens were positive above the upper limit of quantification.	Amb initially and Itraconazole thereafter	LTF

R, recovered; NR, not revealed; LTF, lost to follow up; OC, outcome.
